# Need for adjuvant radiotherapy in oral cancer: depth of invasion rather than tumor diameter

**DOI:** 10.1007/s00405-022-07561-x

**Published:** 2022-08-01

**Authors:** Cosima E. Riemenschnitter, Grégoire B. Morand, Charlotte S. Schouten, Niels J. Rupp, Panagiotis Balermpas, Thomas Gander, Martina A. Broglie Däppen

**Affiliations:** 1 Department of Otorhinolaryngology-Head and Neck Surgery, University Hospital Zurich, Frauenklinikstrasse 24, 8091 Zurich, Switzerland; 2 Department of Pathology and Molecular Pathology, University Hospital Zurich, Zurich, Switzerland; 3 Department of Radiation Oncology, University Hospital Zurich, Zurich, Switzerland; 4 Department of Cranio-Maxillo-Facial and Oral Surgery, University Hospital Zurich, Zurich, Switzerland

**Keywords:** Neoplasm staging, Radiotherapy, adjuvant, Mouth neoplasms, Carcinoma, squamous cell, Otolaryngology, Neoplasm recurrence, local

## Abstract

**Purpose:**

The 8th edition of the TNM Cancer Staging Manual incorporates depth of invasion (DOI) into the pathologic tumor classification for oral squamous cell carcinoma (OSSC). While deep invading tumors with small tumor diameters (TD) have been categorized as early stage tumors in the 7th edition, they are now upstaged, potentially influencing the decision to initiate adjuvant radiotherapy (RT).

**Methods:**

OSCC patients surgically treated with curative intent between 2010 and 2019 were consecutively included. Tumors were staged based on TD only (according to the 7th edition TNM Cancer Staging Manual), then restaged based solely on DOI.

**Results:**

Of the 133 included patients, 58 patients (43.6%) had a different pT-stage when using DOI instead of TD for staging (upstaging in 23.3%). Overall survival (OS) was significantly worse in patients who were upstaged with DOI. In addition, stratification by adjuvant RT showed significant worse OS in upstaged patients without receiving adjuvant RT.

**Conclusions:**

DOI seems to be an import indicator for adjuvant RT in OSCC-patients.

**Supplementary Information:**

The online version contains supplementary material available at 10.1007/s00405-022-07561-x.

## Introduction

The therapeutic standard of care for oral squamous cell carcinoma (OSCC) is surgical treatment with wide local excision of the primary tumor (≥ 1 cm), pathological assessment of the neck (e.g., sentinel node biopsy and/or neck dissection) and reconstruction if required. Adjuvant radiotherapy (RT) is indicated after resection of pT4 tumors and/or if ≥ 2 neck nodes are positive. For pT3 and some pT2 tumors with adverse features, adjuvant RT may be considered [1, 2].

The American Joint Committee on Cancer (AJCC)/International Union Against Cancer (UICC) TNM staging system is a tool to stage cancer according to the anatomic extent of disease [3–8]. Staging helps stratifying patients into treatment- and prognostic groups. The first joint edition of the AJCC/UICC TNM system was published in 1987. Over time, advances in diagnostics and treatment, as well as understanding the prognosis, resulted in various changes.

**Fig. 1:**
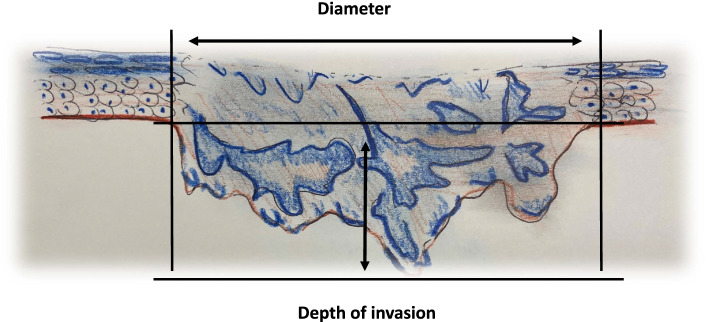
Depth of invasion and tumor diameter measurements as used in the AJCC 8th edition guidelines and the College of American Pathologists

The 8th edition represents the most significant changes to OSCC staging since the first edition of the AJCC Staging Manual: an important prognostic factor in OSCC turned out to be the depth of invasion (DOI) of the primary tumor (Fig. 1). Deep invading tumors are consistently associated with higher risk of occult and clinically apparent nodal metastasis [9, 10]. This led to the integration of DOI in the 8th edition of the TNM system [7, 11–14]. The previous 7th edition was solely based on tumor diameter (TD) to classify T1 to T3 tumors, whereas in the 8th edition both TD and DOI lead to a specific T-classification [6, 15–18]. Furthermore, in 8th edition T4a no longer incorporates extrinsic muscle involvement, which is taken to account in the depth of invasion in T stages 1–3 [19]. After the first release in 2017, UICC/AJCC introduced two further modifications. In the first, a DOI > 20 mm was added to stage a tumor as pT4a. In the second revision, tumors larger than 4 cm and with DOI > 10 mm were shifted to pT4a (Supplemental Table 1). [20]

After the introduction of the 8th TNM classification, the decision to treat with adjuvant RT changed fundamentally and, therefore, also the patient group who received adjuvant RT changed. However, it remains unclear which parameter (TD or DOI) is the most important indicator for adjuvant treatment. Adjuvant RT for superficial pT3 tumors may be overtreatment, causing acute and chronic side effects. Skin damage, lymphedema (significantly more pronounced after multimodal therapy), mucositis and dysphagia associated with significant weight loss as well as dys-/ageusia, fibrosis, xerostomia and in rare cases osteonecrosis can occur [21–25]. On the other hand, the lack of adjuvant RT for deep invading pT1 or pT2 tumors may lead to poor locoregional control rates. In current clinical practice, the side effects of adjuvant treatment are multidisciplinary weighed against the gain in locoregional control.

The aim of this study was to determine whether TD or DOI indicates the need for adjuvant RT. Retrospectively, we took advantage of the TNM classification system change and, therefore, the difference in adjuvant RT decision making, to investigate the relevance of TD and DOI with respect to adjuvant radiotherapy and clinical outcome (histological cross over study).

## Materials and methods

### Study population

After Ethics Review Board approval (protocol number 2016-01799, including amendment dated December 14th, 2018), we retrospectively included all consecutive OSCC patients treated from 2010 to 2019 at the Department of Otorhinolaryngology-Head and Neck Surgery and Department of Cranio-Maxillo-Facial and Oral Surgery of the University Hospital Zurich, Zurich, Switzerland. We excluded patients who received treatment without curative intent, patients with distant disease at diagnosis (M1) or a T4b tumor. All patients were surgically treated with wide local excision, pathological assessment of the neck (sentinel lymph node biopsy or/and neck dissection) and reconstruction if needed after presentation and discussion at the local interdisciplinary tumor board. The need for adjuvant radio(chemo)therapy was discussed in our tumor board after the final pathology was reviewed, according to the NCCN Guidelines in use at this time [26]. Postoperative RT was recommended, if pT4a or/and ≥ 2 lymph node metastasis were present. For pT3 and some pT2 tumors with adverse features [i.e., presence of perineural spread, lymphovascular invasion, close resection margins and/or poorly differentiated (G3) histology], postoperative RT was considered. These considerations remained the same after the introduction of the 8th edition, but because DOI was implemented in the pT-stage, the patients who received adjuvant RT changed. Adjuvant RT comprised an average 66 Gray (Gy) in 2 Gy/fraction delivered to the primary tumor and elective nodal regions received a dose of 50–66 Gy. In case of concomitant chemotherapy (presence of extranodal extension/soft tissue metastasis and/or irradical resection), patients received cisplatin-based chemoradiotherapy (CRT).

During follow-up, patients were regularly physically examined according to our standard head-and-neck-oncology protocol by a multidisciplinary head and neck oncology team. In the first 2 years every 3 months, with additional MRI or PET–CT scanning at 3-, 9- and 15-month post-treatment. Additional investigations during follow-up were performed at the discretion of the attending physician.

### Study design

We reviewed medical records for detailed baseline demographic and clinical data on age, gender, smoking, drinking habits, pathological T-stage, TD, DOI and N-Stadium (Table 1). The DOI was already standardly recorded in our pathology report between 2009 and 2017. Furthermore, we obtained outcome data on local and regional recurrence, distant metastasis, recommended adjuvant therapy and disease-specific and overall survival.

**Table 1:** Patient demographics and clinical characteristics

Characteristic	All patients (*N* = 133)
Age at diagnosis (years)	
Mean (SD)	66 (13.1)
Gender	
Male	
No. (%)	82 (61.7%)
Female	
No. (%)	51 (38.3%)
Tumor subside	
Tongue	
No. (%)	54 (40.6%)
Floor of the mouth	
No. (%)	41 (30.8%)
Upper-/lower gum	
No. (%)	25 (18.8%)
Retromolar trigone	
No. (%)	7 (5.3%)
Buccal	
No. (%)	6 (4.5%)
Smoking	
Yes (%)	78 (58.6%)
No (%)	55 (41.4%)
Alcohol	
Yes (%)	56 (42.1%)
No (%) TD (mm)	77 (57.9%)
Median (IQR)	25 (15–38)
DOI (mm)	
Median (IQR)	10 (4–14)
pT-stage 7th edition	
T1 (< 2 cm)	
No. (%)	44 (33.1%)
T2 (2–4 cm)	
No. (%)	44 (33.1%)
T3 (> 4 cm)	
No. (%)	13 (9.7%)
T4a	
No. (%)	32 (24.1%)
pT-stage 8th edition (*N* = 133)	
T1 (< 5 mm)	
No. (%)	42 (31.6%)
T2 (5–10 mm)	
No. (%)	29 (21.8%)
T3 (> 10 mm)	
No. (%)	44 (33%)
T4a	
No. (%)	18 (13.6%)
Nodal status	
pN0	
No. (%)	82 (57.7%)
pN +	
No. (%)	60 (42.3%)
ENE	
No. (%)	20 (14.1%)
R-status	
R0—resection	
No. (%)	128 (96.2%)
R1—resection	
No. (%)	5 (3.8%)
Postoperative radiotherapy	
Yes (%)	71 (53.4%)
No (%)	62 (46.6%)
Concomitant chemotherapy	
Yes (%)	23 (17.3%)
No (%)	110 (82.7%)

*T* Test for normally distributed variables (age). Mann–Whitney *U* test for non-normally distributed variables (DOI, TD), two-sided Pearson chi-squared test for categorical variables

*ICR* interquartile range, *No.* number, *SD* standard deviation, *TD* tumor diameter, *DOI* depth of invasion

We staged all patients two times, first using the TNM 7th edition and second using DOI only (reduced TNM 8th edition). For staging with DOI only, T1 corresponded to a DOI < 5 mm, T2**:** between 5 and 10 mm and T3**:** > 10 mm [6, 7]. T4a is defined by tumor infiltration into surrounding structures (cortical bone of the mandible or maxilla, facial skin, extrinsic tongue musculature) for both staging systems.

### Statistical analysis

Statistical analyses were performed using SPSS^®^ 23.0.0.0 software (IBM^®^, Armonk, NY, USA). The level of significance was set at *p* < 0.05. For continuous variables, distribution was evaluated for normality according to Gauss’ theorem. For normally distributed variables, mean and standard deviations are given and comparison among study groups was done using the *t* test. For non-normally distributed variables median, interquartile range (IQR) are given. To compare distribution of variables among groups, we used *t* tests and non-parametric Mann–Whitney *U* tests.

We studied three different survival outcomes: time from diagnosis (1) to death (OS) (2) to tumor-related death (disease-specific survival = DSS) and (3) to tumor recurrence (recurrence free survival = RFS). For these three outcomes, we estimated survival curves using Kaplan–Meier. First, we compared survival curves between staging groups classified by TNM 7th edition using log-rank tests. After, we did the same to compare staging groups classified by the reduced TNM 8th edition. In a next step, we grouped all patients into three groups: (1) patients who were down staged using the reduced 8th edition (e.g., 3 cm wide tumor with 4 mm depth of invasion: T2 → T1), (2) patients equally staged (e.g., 3 cm wide tumor with 7 mm depth of invasion: T2 → T2) and (3) patients who were upstaged (e.g., 3 cm wide tumor with 11 mm depth of invasion: T2 → T3). We analyzed the outcomes of the three groups with and without stratification by whether the patient received adjuvant RT.

## Results

### Study population

Of the 142 potentially eligible patients, 9 were excluded, because not all histological parameters were available (DOI). We, therefore, included 133 patients with available TD and DOI. This group did not significantly differ from the total eligible patient cohort and was, therefore, representative. Details on patients, tumor and treatment characteristics are outlined in Table 1. 122 patients (86%) were included before 2017 and were, therefore, staged with the 7th TNM Cancer Staging Manual, of which 69 patients (56.6%) received adjuvant RT. After 2017, 20 patients (14%) were staged in accordance with the 8th TNM Cancer Staging Manual, of which 8 patients (40%) received adjuvant treatment. Hence, a total of 77 patients (54.2%) were treated with adjuvant RT.

The median follow-up time of the analyzed patient cohort was 24 months (range 5–120 months). Thirty-eight of 142 patients (26.8%) developed recurrent disease; in 18 patients (12.7%) local recurrence occurred, in 20 patients (14.1%) regional recurrence and seventeen patients (12.0%) developed distant metastasis. Thirty-four patients (24%) died after a median of 24 months, of which 18 patients died of tumor (disease specific cause).

### Reclassification of pT-stage from TD to DOI

**Fig. 2:**
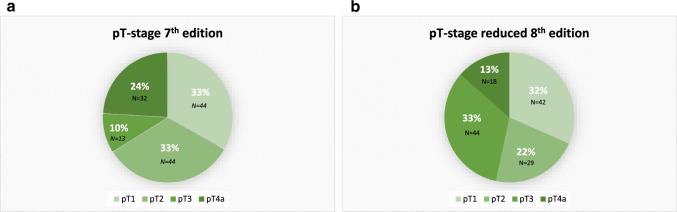
Overview of the distribution across pT-stages for the 7th edition (**a**) and for the reduced 8th edition (**b**)

Figure 2 gives an overview of the distribution across pT-stages for the 7th edition (Fig. 2a) and for the reduced 8th edition (Fig. 2b). A change in the distribution of T-stages is seen: 66% of the patient cohort classified by TD has a low T-stage (T1/T2), whereas the percentage of patients with a low T-stage after reclassifying defined by DOI was 33%. In total, 58 patients (43.6%) had a different T-stage after restaging; 27 patients (20.3%) were down-staged from the 7th TNM edition to the reduced 8th edition, 75 patients (56.4%) were equally staged and 31 (23.3%) were upstaged.

**Fig. 3:**
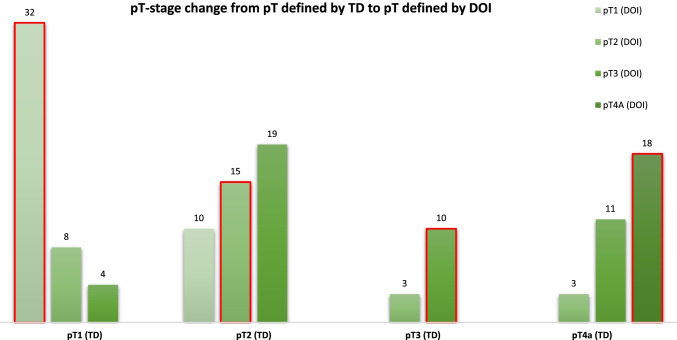
Bar chart shows the four pT-stage groups according to TD on the horizontal axis, divided into the pT-stages after restaging according to DOI. The bars highlighted in red correspond to the group of patients who retained the same pT after restaging (TD→DOI)

Figure 3 shows the four pT-stage groups according to TD on the horizontal axis, divided into the pT-stages after restaging according to DOI. For example, a total of 44 patients were staged as pT2 according to TD, are divided into 10 patients with pT1, 15 patients with pT2 and 19 patients with pT3 after restaging according to DOI.

### Survival outcomes

In Table 2, we compared OS after 24 months between two cohorts (TD vs DOI) for the different T-stages. In general, we found worse OS in the higher T-stages in both groups (TD and DOI). We split both cohorts into patients treated with adjuvant treatment vs patients without adjuvant treatment. In patients with a T1 tumor (in TD and DOI) the OS was lower with adjuvant RT.

**Table 2:** Comparison of the overall survival after 24 months in tumor diameter vs depth of invasion group

	OS T1		OS T2		OS T3		OS T4	
	%	*N*	%	*N*	%	*N*	%	*N*
pT (TD)	96%	(44)	88%	(44)	64%	(13)	67%	(32)
No adj. RT	93%	(35)	65%	(22)	-	(2)*	66%	(6)*
Adj. RT	88%	(9)	78%	(22)	52%	(11)	54%	(26)
pT (DOI)	94%	(42)	79%	(29)	59%	(44)	51%	(18)
No adj. RT	96%	(36)	73%	(15)	51%	(9)	-	(2)
Adj. RT	83%	(6)	83%	(14)	66%	(35)	57%	(16)

*Adj.* adjuvant, *DOI* depth of invasion, *N* number of patients, *OS* overall survival 24 Mt post-therapeutic, *RT* radiotherapy, *TD* tumor diameter

*Four patients refused to do radiotherapy, four patients had cancer in the past on a different location and were already treated with radiotherapy, re-radiation was not possible

Patients with a pT2 and pT3 tumor (in both TD and DOI) showed a better OS when stratified by adjuvant RT: the group with adjuvant treatment showed higher OS than the group without adjuvant RT. For T3/T4 as expected, according to treatment guidelines, the group of patients who did not receive adjuvant radiotherapy was very small. No statistically well-founded statement on survival could be made.

**Fig. 4:**
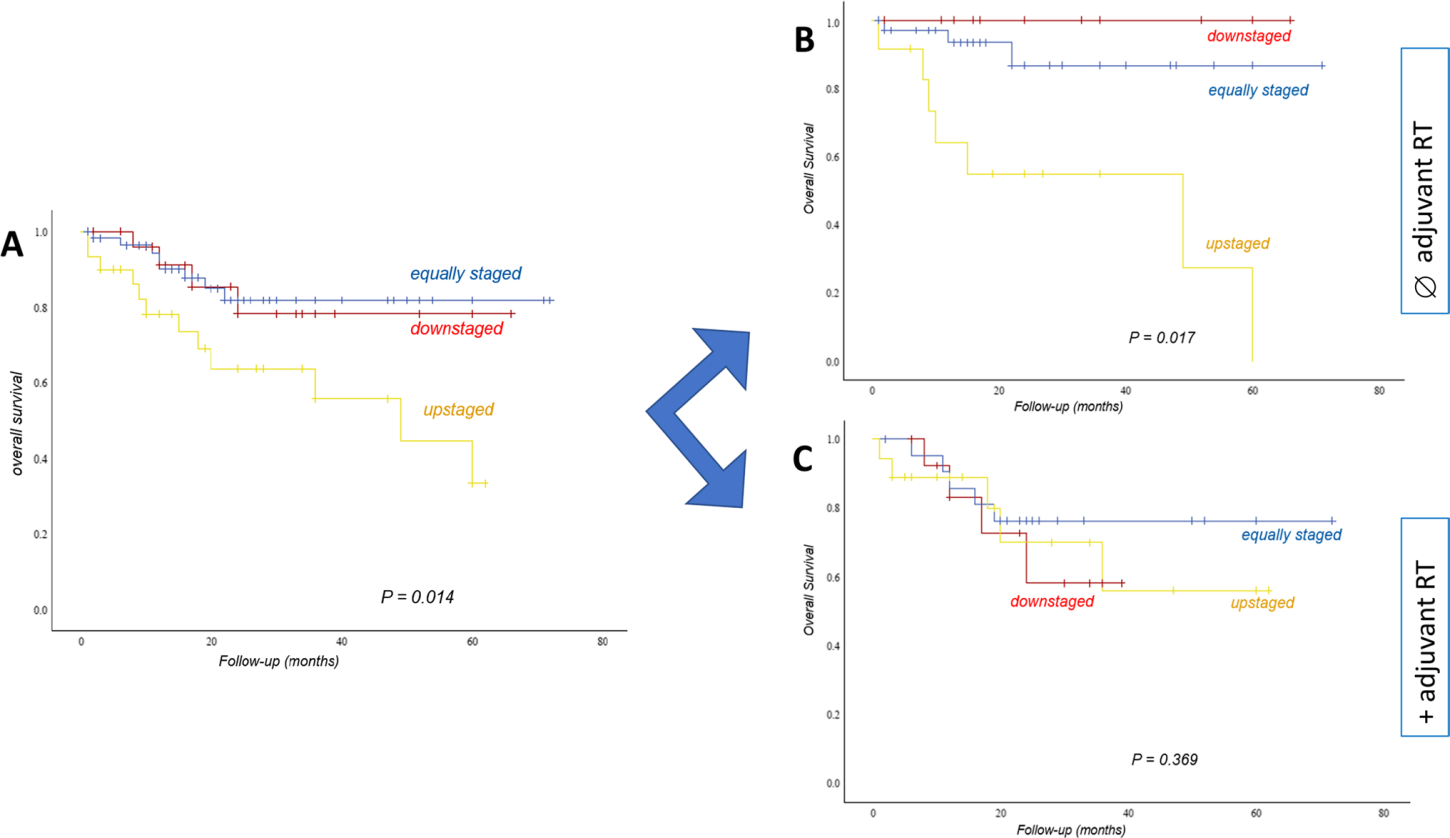
Kaplan–Meier analysis showing significant differences in overall survival when dividing the cases in three groups: equally staged vs down staged vs upstaged after reclassification from 7th TNM edition to DOI only (Panel **A**, log-rank, *p* = 0.014). Then the group of patients with adjuvant treatment was compared with those without postoperative radio(chemo)therapy. The overall survival of the upstaged group without adjuvant RT was significantly worse (Panel **B**, log-rank, *p* = 0.369; Panel **C**, log-rank, *p* = 0.017)

In Fig. 4, the OS for the three different subgroups (upstaged, equally staged, down staged) is shown: OS was significantly worse in patients who had been upstaged with DOI (Fig. 4a, *p* = 0.014). Stratification by whether postoperative RT was performed, showed a significantly worse OS in patients who had been upstaged without receiving postoperative RT (Fig. 4b, *p* = 0.017). In patients with adjuvant RT there was no significant difference in OS between the groups (Fig. 4c, *p* = 0.369). For DSS and RFS we did not find significant differences between the three groups.

## Discussion

Patients with oral cancers are primarily treated with a surgical approach and postoperative RT is considered in the interdisciplinary tumor board in case of pT2/pT3 tumors with adverse features. The 8th edition of the AJCC staging manual includes DOI of the primary tumor into the pT-stage. However, it is unclear if DOI indicates the need for adjuvant RT in OSCC patients. This study shows that 44% of the patients are differently staged when using DOI only instead of TD to define T-stage and a shift is seen from 34% high-staged patients (T3–T4, staged with TD) to 67% high-staged patients (staged with DOI). In addition, OS is significantly lower in upstaged patients without adjuvant RT.

Figure 2B highlights, that patients who were upstaged using DOI and did not receive adjuvant RT did worse than their counterpart without upstage or those receiving adjuvant RT. This indicates that deep DOI that stayed “out of consideration” at the time of indicating adjuvant RT was detrimental to survival.

In high-risk situations such as non-in-sano resection (R1/R2) or extracapsular extension adjuvant RT with concomitant chemotherapy if possible was applied and is still recommended regardless of the DOI or the T-category. [27, 28] On the other hand, the classical intermediate risk situations which were used for adjuvant RT indication were pT3 or pT4 tumors, one single node > 3 cm (pN2a in UICC version 7.0) or more than one involved lymph node (pN2b–pN2c in UICC version 7.0) [29–31]. After the implementation of DOI in the definition of T-category this absolutely conforms with the observed risk situation, as a tumor with DOI of 10 mm automatically qualifies for adjuvant RT as pT3, which was not the case before. In the past this was decided on individual level and physicians in the tumor conference always asked this additional information to see if a patient with a pT2 tumor (old classification) could benefit from adjuvant RT in case of deep invasion. Finally, in case of 3 or more “minor” risk factors, such as pN1 (UICC version 7.0), perineural invasion (pn1), lymphatic invasion (L1), vascular invasion (V1), we recommend adjuvant RT in our center, but this is strictly depending on the individual age, performance status and wish of the patient and varies amongst institutions. In the past a DOI of 5 mm or more was frequently added to these “minor” risk factors.

Fridman et al. focused on the role of adjuvant RT in early stages of oral cavity cancer. The study was histologically evaluated according to the 7th edition of the American Joint Committee on Cancer System. In the performed multivariate analysis, no increased risk of local recurrence was found with regard to the depth of invasion, while close resection margins were associated with an increased risk of recurrence. At first sight this seems to contradict the results of our study. However, it is known that there is an increased likelihood of narrow deep resection margins in deep invading tumors. In their study, close resection margins might have correlated with DOI, and thereby responsible for higher risk of recurrence [32–36].

Narayana et al. wrote a review about adverse pathologic features in early OSCC and the role of adjuvant RT in 2017 after the introduction of 8th edition of the American joint cancer classification. They enrolled adverse pathologic features in early stage oral cancer to detect a subgroup of patients who would profit from adjuvant RT [37]. Based on their evidence, the authors recommended to decide to administer adjuvant therapy on an individual basis; patients with > 1 adverse pathological features are likely to benefit from adjuvant RT and the use of risk-scoring systems may help in decision making.

Several limitations of our single center study need to be mentioned. First, we are a low volume center and had a relatively small number of patients, some of which had to be excluded from the study, because relevant histological parameters were not documented. Second, two different TNM classifications (7th and 8th edition) were used and this led to inconsistency in the application of adjuvant RT. However, we used this inconsistency in our study population to perform a historical cross-over study: we were able to stage each patient twice (with TD and with DOI-only) and showed changes in pT-staging. Finally, an equally important but subtler bias may be the Will Rogers phenomenon. This term was described by Feinstein et al., who often quoted a Will Rogers joke that “when the Okies moved to California, the IQ of both states went up.” This phenomenon of stage migration/upstaging can occur when patients are reclassified, as often happens after the introduction of more sensitive staging tools or changes in classification systems and has been shown among men with newly diagnosed lung cancer as well as in breast cancer patients [38–40].

## Conclusion

We found an upstaging rate of 23.3% when using DOI instead of TD. Interestingly, upstaged patients showed worse survival rates when treated by surgery alone, while the application of adjuvant RT led to comparable survival estimates. Our results support DOI as an important prognosticator and more adequate parameter to stage oral cavity cancer patients and to indicate adjuvant radiotherapy.

## Supplementary Information

**Additional file 1: Supplemental Table 1.** Pathologic T-stages according to the TNM 8th edition and its modifications (01/2018 and 06/2018).

## Data Availability

The data sets used and/or analysed during the current study are available from the corresponding author on reasonable request.
