# Supplementary material for: Need for adjuvant radiotherapy in oral cancer: depth of invasion rather than tumor diameter
Source: Eur Arch Otorhinolaryngol. 2022 Aug 1;280(1):339–46. doi: 10.1007/s00405-022-07561-x (PMC9813178; doi:10.1007/s00405-022-07561-x)
Supplement: Supplementary file 1 — Additional file 1: Supplemental Table 1. Pathologic T-stages according to the TNM 8th edition and its modifications (01/2018 and 06/2018). [file 405_2022_7561_MOESM1_ESM.docx]

**Supplemental Table 1.** Pathologic T-stages according to the TNM 8th edition and its modifications (01/2018 and 06/2018)

|  | - TNM 8^th^ (2017) | - Modification 01/2018 | - Modification 06/2018 |
| --- | --- | --- | --- |
| - T1 | - ≤2 cm TD - ≤5 mm DOI | - No change | - No change |
| - T2 | - ≤2 cm, DOI >5 mm but ≤10 mm or >2 cm but ≤4 cm, DOI ≤10 mm | - No change | - ≤2 cm, DOI >5 mm or - >2 cm but ≤4 cm, DOI ≤10 mm |
| - T3 | >4 cm or any tumor with  DOI > 10 mm | - >4 cm or any tumor with DOI >10 mm but ≤20 mm | - >2 cm and ≤4 cm with DOI >10 mm or >4 cm and DOI ≤10 mm |
| - T4a | - Tumor invades adjacent structures only | - Tumor invades adjacent structures only or - Extensive tumor with bilateral tongue involvement or - >4 cm with DOI >20 mm | - Tumor invades adjacent structures only or   >4 cm with DOI >10 mm |
| - T4b | - Tumor invades masticatory space, pterygoid plates or skull base or encases internal carotid artery | - No change | - No change |
